# Aging-related decline in the neuromotor control of speech production: current and future

**DOI:** 10.3389/fnagi.2023.1172277

**Published:** 2023-04-20

**Authors:** Huijing Hu, Jingting Li, Sixuan He, Yan Zhao, Peng Liu, Hanjun Liu

**Affiliations:** ^1^Institute of Medical Research, Northwestern Polytechnical University, Xi'an, China; ^2^Department of Rehabilitation Medicine, The First Affiliated Hospital, Sun Yat-sen University, Guangzhou, China; ^3^Guangdong Provincial Key Laboratory of Brain Function and Disease, Zhongshan School of Medicine, Sun Yat-sen University, Guangzhou, China

**Keywords:** speech production, aging, neuromotor control, auditory feedback, voice

## Introduction

Speech production is a complex, neuromotor behavior that involves the physiological, neurological, respiratory, and muscular systems. The decrement in overall function of these systems occurs to advancing age, leading to aging-related changes in speech production. Great efforts have been made to investigate the acoustic changes in the aging speech in the past few decades. For example, changes with aging in voice fundamental frequency (*f*_o_) occur through adult life (Ramig and Ringel, 1983; Decoster and Debruyne, 1997; Mueller, 1997; Sataloff et al., 1997). When compared to younger adults, older adults exhibit greater voice *f*_o_ instability and lower vowel formants (Gorham-Rowan and Laures-Gore, 2006; Torre and Barlow, 2009). Also, decreased speaking rates (Duchin and Mysak, 1987; Wohlert and Smith, 1998) and speech accuracy (Sadagopan and Smith, 2013; Bilodeau-Mercure et al., 2015) represent substantial aging-related deficits in prosody and articulation.

In addition to the acoustic changes in the aging speech, the ability to monitor and correct acoustic changes during speech production that depends on the integration of auditory feedback and motor systems, or speech motor control, is also compromised by normal aging. By unexpectedly perturbing voice *f*_o_ in auditory feedback during ongoing vocalization, for example, several studies found larger and more variable compensatory vocal responses produced by older adults than by younger adults (Liu et al., 2010; Liu P. et al., 2011; Li et al., 2018). In accordance with abnormally larger vocal compensate responses to pitch perturbations in patients with neurological disorders relative to healthy controls (Liu et al., 2012; Huang et al., 2016; Mollaei et al., 2016; Ranasinghe et al., 2017), enhancement of vocal compensations for perturbed auditory feedback observed in older adults may reflect a decline in speech motor control with normal aging.

In contrast to aging-related changes in speech acoustics, the neuromotor control of speech production with advancing age has received much less attention. Investigations of speech motor control in aging adults can provide significant insights into our understanding of the developmental changes in speech production across the adult lifespan. Speech aging has been attributed to age-related changes in the peripheral and neurological systems and cognitive functions (Ramig et al., 2001; Tucker et al., 2021) as well as sex difference (Torre and Barlow, 2009). Therefore, this review discusses multiple factors that are involved in aging-related neuromotor control of speech production, including laryngeal physiology, brain structure and function, higher-order cognitive functions, and sex-aging interaction.

### Laryngeal physiology

Aging-related laryngeal structures accompany with physiological changes, such as decrease and degeneration in thyroarytenoid muscle fiber diameter (Ramig et al., 2001), decreased firing rates (Baker et al., 1998), larger internal stiffness as well as greater mass of vocal folds (Gorham-Rowan and Laures-Gore, 2006). Notably, groups of laryngeal muscles have been identified to be involved in regulating voice *f*_o_ by controlling the geometry and tension of the vocal folds (Hirano et al., 1970; Ludlow et al., 1992), and our work specified significant contributions of laryngeal cricothyroid and thyroarytenoid muscles to compensatory vocal adjustment (Liu H. et al., 2011). Since the ability to precisely control vocal production declines in older adults (Ballard et al., 2001), excessive vocal compensations when facing pitch errors observed in aging adults (Liu et al., 2010; Liu P. et al., 2011; Li et al., 2018) may be attributed to their deficits in controlling laryngeal muscles to produce the desired speech sounds. Alternatively, aging-related decline in speech production may be related to changes in the use of feedback control strategies with advancing age. Successful control of speech production requires a dynamic balance of auditory and somatosensory feedback (Golfinopoulos et al., 2010), and deficits in somatosensory feedback shifts the balance to auditory feedback (Lametti et al., 2012). Larson et al. (2008) found enhanced vocal compensations for pitch errors induced by anesthetization of the vocal folds when compared to normal kinesthesia, suggesting increased reliance on auditory feedback due to interfered somatosensory feedback. Given impaired kinesthetic function caused by aging-related laryngeal changes (Ramig et al., 2001), older adults may rely more on auditory feedback such that they are more susceptible to errors in vocal output, resulting their reduced accuracy in the neuromotor control of vocal production.

### Brain structure and function

In addition to the peripherical mechanisms, structural and functional changes in the aging brain also contribute to the age-related decline in speech production (Eckert et al., 2008; Harris et al., 2009; Soros et al., 2011; Tremblay et al., 2013, 2017; Tremblay and Deschamps, 2016). When compared to younger adults, for example, older adults exhibited longer speech movement time that was significantly correlated with their structural changes in the bilateral anterior insula, bilateral striatum, rostral supramarginal gyrus, left primary motor area and right inferior frontal sulcus (Tremblay and Deschamps, 2016). As well, older adults showed greater activation in the inferior frontal gyrus, precentral gyrus, anterior insula, and supplementary motor area during overt speech production than younger adults (Soros et al., 2011). In addition, subcortical structures such as the basal ganglia and cerebellum are also involved in the neuromotor control of vocal production, as evidenced by abnormally enhanced vocal compensations for pitch perturbations in aging patients with Parkinson's disease (PD; Liu et al., 2012; Chen et al., 2013; Huang et al., 2016; Mollaei et al., 2016) and spinocerebellar ataxia (SCA; Parrell et al., 2017; Houde et al., 2019; Li et al., 2019).

Note that there is a significant decrease in the number of inhibitory synapses with advancing age (Kovacevic et al., 2005) that limit the capacity to suppress responses to repetitive auditory stimuli (Amenedo and Diaz, 1998), which may be responsible for overcompensation for vocal pitch errors (Liu et al., 2010; Liu P. et al., 2011; Li et al., 2018) and increased cortical responses to pure tones (Stephen et al., 2010) in older adults. On the other hand, previous studies on speech perception has shown aging-related increase in the latency of neural responses to auditory stimuli (Geal-Dor et al., 2006; Matilainen et al., 2010), which has been attributed to the neuronal loss in the aging brain (Jernigan et al., 2001). This delayed temporal processing of speech sounds has been characterized by prolonged event-related potential (ERP) N1 and/or P2 responses in latency in older adults (Tremblay et al., 2003; Martin and Jerger, 2005), which may help explain older adults' slower P2 responses to auditory feedback errors during vocal pitch regulation compared to younger adults (Li et al., 2018).

### Higher-order cognitive functions

Previous research has shown that speech changes in older adults only can be partially explained by physiological changes to the speech system (Bilodeau-Mercure and Tremblay, 2016). Cognitive decline in relation to working memory, attentional control, and executive function during aging (Salthouse, 1996; Park et al., 2002) has an impact on language/speech production (Barker et al., 2020). Aging-related declines in working memory, for example, result in the production of inaccurate speech sounds (Sadagopan and Smith, 2013). Inhibitory control deteriorates during aging (Nielson et al., 2002), and deficits of this function have been attributed to off-topic speech in older adults (Gold and Arbuckle, 1995). Also, evidence from people with cognitive impairment suggests a link between cognitive function and speech production. For example, PD patients with motor speech disorders were associated with greater attention/memory dysfunctions (Liu et al., 2019), and individuals with mild cognitive impairment exhibited weaker voice quality and slower articulation rates than healthy controls (Themistocleous et al., 2020). Also, Alzheimer's disease (AD) patients exhibited enhanced and prolonged vocal compensations for pitch perturbations that were significantly related to their dysfunctions in working memory and executive control (Ranasinghe et al., 2017).

A top-down inhibitory mechanism that has been proposed to address the neuromotor control of vocal production from a cognitive perspective (Guo et al., 2017; Ranasinghe et al., 2017; Liu et al., 2020) may account for aging-related changes in vocal motor control. Specifically, the left dorsolateral prefrontal cortex (DLPFC) is crucially involved in inhibitory control (Burle et al., 2004), and decreased activity in this region leads to impaired inhibitory control processes (Barber et al., 2013). On the other hand, decreased activity in the left DLPFC was significantly correlated with excessive vocal compensations for pitch perturbations in AD patients (Ranasinghe et al., 2017). Moreover, inhibitory continuous theta burst stimulation (c-TBS) over left DLPFC led to enhanced compensations for vocal pitch errors (Liu et al., 2020). These findings suggest the existence of a top-down inhibitory mechanism mediated by the left DLPFC that underlies auditory-motor control of vocal production, by which the audio-vocal system monitors and corrects feedback errors to produce the desired speech sounds with stability and precision. According to the inhibition deficit hypothesis, deficient inhibitory control occurs to older adults so that they fail to suppress task-irrelevant information (Campbell et al., 2020). Therefore, overcompensation for vocal feedback errors accompanied by decreased cortical activity observed in healthy older adults (Liu P. et al., 2011; Li et al., 2018) and patients with cognitive impairment (Ranasinghe et al., 2017, 2019) can be conceivably attributed to impairment of this top-down mechanism that fails to produce inhibitory control over vocal motor behaviors. These findings support the idea that speech motor control should be viewed as a “cognitive-motor accomplishment” (Kent, 2000), although the precise role of higher-order cognitive functions for speech motor control in older adults is still far from clear.

### Sex and aging interaction

During the aging process, the substantial sex differences in speech acoustics are important but often overlooked. For example, men decrease their voice *f*_o_ slightly until 50 years of age and then increase it afterwards, which is in contrast with women whose voice *f*_o_ continuously decreases or stays constant until menopause and then decreases (Sataloff et al., 1997; Torre and Barlow, 2009). Our work reveals an aging-related, sex-specific changes in speech motor control, as reflected by decreased P2 amplitudes with aging in men only and men producing increased N1 and P2 responses than women were found in young adults but not in elderly adults (Li et al., 2018). These sex-related changes in speech production with aging have been accounted for by aging-related differences in the laryngeal and brain structures and functions between men and women. For instance, throughout adulthood, women experience more pronounced laryngeal drops and lengthening of the vocal tract than men, leading them to adjust their vowel and speech acoustic differentially to accommodate these changes (Rastatter and Jacques, 1990; Linville and Rens, 2001). Progressive changes in brain structure and function also differ between men and women (Cowell et al., 1994; Kakimoto et al., 2016), as evidenced by greater age-related increase in lateral fissure cerebrospinal fluid volume and greater variation in frontal cortex atrophy in men than in women (Coffey et al., 1998; Kakimoto et al., 2016).

More importantly, both animal and human studies have shown the impact of sex hormonal levels throughout the menstrual cycle or after menopause on controlling vocal production (Walpurger et al., 2004; Wadnerkar et al., 2006; Caras, 2013; Zhu et al., 2016). Specifically, in one study on young women across the menstrual cycle, Zhu et al. (2016) showed that larger vocal compensations for pitch perturbations were correlated with lower levels of estradiol concentration significantly and smaller P2 responses were associated higher levels of progesterone concentration. Although the underlying mechanisms remain largely unknown, sex hormones may modulate vocal motor behavior by activating receptor-coupled effector mechanisms through binding to specific hormone receptors that exist in the larynx and brain (Newman et al., 2000; Goldstein et al., 2001; Gheller et al., 2016). Taken together, these findings shed light on the substantial differences between men and women in elucidating how the aging process affects speech motor behaviors.

### Future research

Clarifying the effects of aging on speech motor control not only provides insights into the relationship between the dynamics underlying speech production and neurobiology of aging, but also has important enlightenments for the treatment of motor speech disorders that frequently occur to aging adults. Future work is warranted to investigate the neural mechanisms underlying the effects of aging on speech motor control. On the other hand, abnormal vocal compensation for perturbed auditory feedback has been suggested to be a potential behavioral index of declined or impaired speech motor control (Ranasinghe et al., 2017; Li et al., 2018, 2021). More recently, non-invasive brain stimulation (NIBS) such as continuous theta burst stimulation (cTBS) has been applied over specific brain regions of aging patients with PD and SCA (Dai et al., 2022; Lin et al., 2022), resulting in a normalization of their overcompensation for vocal pitch perturbations. Therefore, NIBS may be a promising strategy that not only improves aging-related decline in the neuromotor control of speech production but also provides novel treatment of motor speech disorders that occur to aging patients with neurological disorders. Further investigations, however, are required to explore this topic in the future.

## Author contributions

HH and HL: conceptualization. HH and JL: writing—original draft preparation. HH, SH, YZ, PL, and HL: writing—review and editing. All authors contributed to the article and approved the submitted version.
